# Knowledge, attitude and practices regarding COVID-19 and their associated factors in patients with type 2 diabetes attending Abdullah-Khalil diabetes center, Omdurman: A cross-sectional study

**DOI:** 10.1097/MD.0000000000032561

**Published:** 2022-12-30

**Authors:** Yousra S. Ahmed, Meirfat I. Mohamed, Elfatih A. Hasabo, Alaa T. Omer, Istabraq I. Abdelgadir, Sara N. Bashir, Noha E. EL hag

**Affiliations:** a Faculty of Medicine, University of Khartoum, Khartoum, Sudan.

**Keywords:** attitude, COVID-19, diabetes mellitus, knowledge, practice, Sudan

## Abstract

Novel corona virus disease 2019 is the major threat for human life nowadays worldwide. This study aims to assess the knowledge, attitude and practices regarding COVID-19 among patients with type 2 diabetes attending Abdullah Khalil diabetes center (Omdurman Teaching Hospital). A facility-based observational descriptive cross-sectional study was carried out between January and February 2021, using non-probability quota sampling technique in Abdullah-Khalil diabetes center via the use of a structured close-ended interview questionnaire. It consisted of 19, 10, and 10 questions pertaining to knowledge, attitude and practices towards COVID-19 respectively. A total of 249 patients with type 2 diabetes were included. Of them, 132 (53.0%) were females and the majority (53.0%) aged between 41 and 60 years old. Concerning their education and marital status, 70 (28.1%) were primary school level and 208 (83.5%) were married. 89.6% of participants reported mass media as main source of information regarding COVID-19. Patients with type 2 diabetes attending Abdullah-Khalil diabetes center have good knowledge, positive attitude and good prevention practices towards COVID-19. Most respondents had good knowledge, positive attitude and good practices as 78.7%, 97.6%, and 69.9%, respectively. Educational level and source of information showed statistically significant association with the knowledge, attitude. However, the practice showed only statistical association with the mass media as a source of information (*P* = .006).

## 1. Introduction

*“Coronavirus disease (COVID-19) is an infectious disease caused by a newly discovered coronavirus named severe acute respiratory syndrome coronavirus2 (SARS-CoV-2).”*^[1]^ Respiratory droplets of an infected person during coughing or sneezing are the primary way of spread. Touching of nose, mouth, or eyes after touching a contaminated surfaces with droplet containing COVID-19 is another mode of transmission. The World Health Organization declared COVID-19 pandemic in March 2020. As of 30 March 2021, and World Health Organization reported more than 127,349,248 confirmed cases with 2787,593 confirmed deaths, globally.^[1]^ Sudanese Federal Ministry of Health reported 30,111 and 2063 confirmed cases and deaths respectively to date March 28, 2021.^[2]^ Up to November 2020, Sudan reported a relatively high COVID-19 case fatality rate (7.7%) from different sates and (4%) in Khartoum particularly.^[3]^

“*Diabetes is a chronic, metabolic disease characterized by elevated levels of blood glucose, which leads over time to serious damage of body organs.”*^[1]^ Diabetes has 2 types. Type 2 diabetes is the most common, and occurs usually in adults. About 422 million people worldwide have diabetes, and majority of them were living in low-income and middle-income countries, with steadily increasing prevalence.^[1]^ Sudan is 1 of 7 countries with a medium prevalence of diabetes (9–12%). Diabetes, hypertension and cardiovascular diseases are considered important risk factors for undesirable outcome in patients diagnosed with COVID-19.^[4]^ Dysfunction of the immune system due to hyperglycemia makes the patients with type 2 diabetes more susceptible to infections.^[5]^ Diabetes mellitus aggravates the severity of COVID-19, wherein COVID-19 leads to worsening of glucose level, as a brutal 2-way interaction.^[6]^ A Chinese observational report of 1099 COVID-19 patients showed that out of 173 patients with severe disease were 28 (16.2%) of them has diabetes as comorbidity.^[7]^ Diabetes is an important comorbidity in COVID-19 patients due to its high prevalence.^[8]^ A study showed that among patients with severe COVID-19, those with diabetes were susceptible to receiving mechanical ventilation, ICU admission and had higher mortality.^[9]^

In Sudan, diabetes mellitus was the second common comorbidity among the Sudanese patients with COVID-19, and it was found significantly associated with disease severity and the final outcome (in-hospital death).^[10]^ Also, another study in Sudan found that COVID-19 patients with diabetes mellitus as comorbidity recorded a higher number of deaths compared with those without diabetes.^[11]^

A study showed a high mortality rate among Sudanese COVID-19 patients (17.6%) and its obvious link with the worsening economic situation, drugs insecurity and the scarcity of medical resources.^[10]^

This represents a burden on our country especially during this period of health and economic systems collapse in Sudan, and thus prevention and reduction of spread is the best solution till the vaccine be affordable or definitive treatment emerged. The increasing number of cases, especially with the beginning of the second wave in Sudan necessitates the importance of assessing the knowledge, attitude and preventive practices towards COVID-19 among patients with type 2 diabetes and explore the effectiveness of various awareness programs on this highly susceptible category.

A Sudanese study showed that (68.3%) of its participants had a good knowledge towards COVID-19 and this significantly associated with good preventive practices.^[12]^

The exploring of gabs in knowledge and commitment to means of protection and precautionary recommendations will help in control of this pandemic. Our research could be used as a baseline to pay attention of health authorities to this category and encourage the raising awareness campaigns for patients with type 2 diabetes in different media and in their regular visits to the diabetes centers. Therefore, we conducted this study to assess knowledge, attitude and practice of COVID-19 among patients with type 2 diabetes attending diabetes center in Sudan.

## 2. Methods

### 2.1. Ethics approval and consent to participate

Ethical approval of the study was obtained from the Technical and Ethical Review board in Department of Community Medicine, Faculty of Medicine, University of Khartoum, Khartoum, Sudan (ethical approval number = 12/2020).

### 2.2. Study design and settings

This observational cross-sectional study was conducted at Abdullah-Khalil diabetes center, Omdurman, Sudan between January and February 2021. The center belongs to Omdurman teaching hospital, which locates in Omdurman locality, Khartoum state, Republic of Sudan.

Departments within Abdullah-Khalil diabetes center were: Specialty clinic, medical laboratory, Pharmacy, Health-insurance office, Accounts office, diabetic-foot clinic, nutrition office.

### 2.3. Participants

We included all adult patients diagnosed with diabetes visiting Abdullah-Khalil diabetes center during the study period (January 31, 2021 till February 28, 2021). Individuals who refused to participate were excluded from the study.

### 2.4. Instruments used to measure the variables of interest in the study

Data were collected using a validated structured close-ended interview questionnaire in a Google form format. The questionnaire was adopted and developed by reviewing previously published similar studies.^[13–15]^ The developed tool was reviewed by senior researcher and professional from Department of Community Medicine, Faculty of Medicine, University of Khartoum. The initial questionnaire was prepared in English then converted into Arabic and revised by the supervisor and a social worker. Before starting data collection, a pilot survey was conducted to test for questionnaire practicality and correct tool errors.

The questionnaire consisted of 4 sections of questions:

The first part: included 8 questions to assess sociodemographic data of the participants and general questions, which were: age, gender, educational level, marital status, familiarity with the disease and the emergency hotlines, the source of information used and if having any other chronic disease.

The second part: included 16 questions to assess the knowledge of patients with type 2 diabetes about COVID-19 facts, symptoms, transmission, and risk in comorbid patient. Knowledge questions answered as yes, no, “I do not know” opinion. The correct answer was assigned 1 point, while incorrect or “I do not know” answer was given 0 point. The fourth negative question was transformed into a positive question. Respondent’s overall knowledge was classified as good, moderate, and poor if the score was between 80% and 100% (12.8–16 points), 60% and 79% (9.6–12.64 points), and <60% (<9.6 points) respectively.

The third part: included 10 questions to determine the attitude of patients with type 2 diabetes towards COVID-19. The tenth negative question was transformed into a positive question. The scores were scored based on the participants’ response to each statement as 1 point for strongly agree or agree, 0 point for neutral, disagree, and strongly disagree. Total scores ranged from 0 to 10, with high scores (>60%) indicating positive attitudes.

The fourth part: included 10 questions to assess the preventive practices of patients with type 2 diabetes towards COVID-19. Answered as yes or no; 1 point for the correct answer and 0 point for the incorrect answer was given. Respondents’ overall practice was classified as good, moderate and poor if the score was between 80% and 100% (8–10 points), 60% and 79% (6–7.9 points), and <60% (<6 points) respectively.

### 2.5. Data collection and sampling

COVID-19 preventive precautions were considered during data collection and dealing with participants. We collected data from all consecutive participants who met our inclusion criteria during the study. We started collecting data daily from 7:00 am till the closure of the center during all working days for this center.

### 2.6. Statistical analysis

Data were cleaned and checked for completeness then entered into a statistical package for social sciences (SPSS) version 23 (SPSS Inc., Chicago, IL) for analysis. The data were analyzed descriptive statistics to summarize the participants’ baseline characteristics, knowledge, attitude, and prevention practices towards COVID-19 and presented by frequency and percentage (%) and mean ± standard deviation (SD). Chi square test and fisher exact test were used to assess the association between the baseline variables with the levels of knowledge, attitude and practice toward COVID-19. The level of *P* value less than .05 was considered as statistically significant for this study.

## 3. Results

### 3.1. Participants’ information

A total of 249 patients with type 2 diabetes participated with a response rate of 100%. 132 (53.0%) of participants were female and the majority of the participants 132 (53.0%) aged between 41 to 60 years old. Among the total study respondents, around 208 (83.5%) were married. Concerning the educational level of the participants, 70 (28.1%) were primary school level. 64 (25.7%) of the participants were hypertensive, 12 (4.8%) had cardiovascular diseases while 10 (4.0%) had pulmonary diseases as comorbidities. All the participants 249 (100%) heard about COVID-19, but only 50 (20.1%) of them know the COVID-19 emergency hotlines for inquire or cases report in Sudan. The mass media used by 223 (89.6%) of the participants as a main source of information regarding the COVID-19 (Table 1).

**Table 1:** Baseline characteristics of patients with type 2 diabetes attending Abdullah-Khalil diabetes center, Omdurman teaching hospital, Sudan (n = 249).

Variables	Overall, N = 249*
Age group	
18–40 yr	52 (20.9)
41–60 yr	132 (53.0)
Above 60 yr	65 (26.1)
Gender	
Male	117 (47.0)
Female	132 (53.0)
Educational level	
Illiterate	46 (18.5)
Khalwa (Holy Quran institute)	12 (4.8)
Primary school	70 (28.1)
Secondary school	69 (27.7)
University level of above	52 (20.9)
Marital status	
Single	19 (7.6)
Married	208 (83.5)
Divorce	11 (4.4)
Widow	11 (4.4)
Do you hear about COVID-19	
Yes	249 (100)
No	0 (0)
Do you know the COVID-19 emergency hotlines for inquire or cases report	
Yes	50 (20.1)
No	199 (79.9)
From where do you get information about COVID-19 (multiple response question)	
Mass media	223 (89.6)
Social media	65 (26.1)
Family/friends	177 (71.1)
Health care professionals	29 (11.6)
Do you have any of the following diseases	
Hypertension	64 (25.7)
Cardiovascular disease	12 (4.8)
Pulmonary disease	10 (4.0)
Level of knowledge about COVID-19	
Poor	17 (6.8)
Moderate	36 (14.5)
Good	196 (78.7)
Level of attitude toward COVID-19	
Positive	243 (97.6)
Negative	6 (2.4)
Level of practice about COVID-19	
Poor	26 (10.4)
Moderate	49 (19.7)
Good	174 (69.9)

* Data were presented as number (%).

### 3.2. Knowledge about COVID-19 among patients with type 2 diabetes

The mean ± SD score was 13.5 ± 3.1 for Knowledge about COVID-19. Most of the respondents (92.8%) stated that COVID-19 is an infectious disease. About 165 (66.3%) of the participants reported that there is a difference between COVID-19 and Cold flu in symptoms and severity. 174 (69.9%) of the respondents indicated that they were aware of the incubation period of the disease as 2 to 14 days. 165 (66.3%) stated that COVID-19 had no specific effective treatment or vaccine currently in Sudan. About 208 (83.5%) of the study respondents distinguished that COVID-19 is caused by a virus. 227 (91.2%) of study respondents knew the main symptoms of COVID-19 like fever, cough and shortness of breath, while 179 (71.9%) knew the less common symptoms like headache, loss of smell or taste and diarrhea (Table 2).

**Table 2:** Responses of knowledge questions about COVID-19 facts, symptoms, transmission and risks in diabetes comorbid patient among patients with type 2 diabetes attending Abdullah-Khalil diabetes center, Omdurman teaching hospital, Sudan (n = 249).

Knowledge-related questions	Responses		
	Yes (N, %)	No (N, %)	I Do not know (N, %)
COVID-19 is an infectious disease	231 (92.8)	3 (1.2)	15 (6.0)
COVID-19 is caused by virus	208 (83.5)	5 (2.0)	36 (14.5)
COVID-19 exists in Sudan	234 (94.0)	5 (2.0)	10 (4.0)
There are no differences between COVID-19 and Common cold in symptoms and severity	55 (22.1)	165 (66.3)	29 (11.6)
The incubation period of the disease is 2–14 d	174 (69.9)	6 (2.4)	69 (27.7)
COVID-19 had no specific effective treatment or vaccine currently in Sudan	133 (53.4)	70 (28.1)	46 (18.5)
Fever, cough and shortness of breath are the main symptoms	227 (91.2)	1 (0.4)	21 (8.4)
There are other symptoms like headache, loss of smell or taste and diarrhea	179 (71.9)	10 (4.0)	60 (24.1)
The prevalence of COVID-19 in Sudan is increasing	199 (79.9)	18 (7.2)	32 (12.9)
The disease is transmitted through contacts with infected person (talking, shaking hand, hugging)	221 (88.8)	9 (3.6)	19 (7.6)
The disease is transmitted through droplets during coughing and sneezing	232 (93.2)	2 (0.8)	15 (6.0)
The disease is transmitted through touching an infected surface with the virus	223 (89.6)	4 (1.6)	22 (8.8)
COVID-19 is more dangerous in elderly people	227 (91.2)	8 (3.2)	14 (5.6)
COVID-19 is more dangerous in patients with chronic disease like diabetes	236 (94.8)	2 (0.8)	11 (4.4)
Wearing face mask reduce the risk of infection	237 (95.2)	3 (1.2)	9 (3.6)
Washing hands with water and soap for at least 20 s or alcohol reduce the risk of infection	241 (96.8)	2 (0.8)	6 (2.4)

* Data were presented as number (%).

The study respondents having information of modes of transmission including contacts with infected person, droplets during coughing or sneezing and touching an infected surface with the virus by 221 (88.8%), 232 (93.2%), 223 (89.6%) respondents, respectively. Moreover, most of the participants 237 (95.2%) knew that wearing face mask reduce the risk of infection, and 241 (96.8%) participants stated that washing hands with water and soap for at least 20 seconds or alcohol reduce the risk of infection. 236 (94.8%) of respondents reported potentially the disease is more dangerous in in patients with chronic disease like diabetes.

Also, 227 (91.2%) of participants knew that the disease is more dangerous in elderly people (Table 2).

### 3.3. The overall level of knowledge about COVID-19 among patients with type 2 diabetes

Data analysis showed that the prevalence of poor knowledge about COVID-19 among the study participants was 6.8% whereas the prevalence of moderate and good knowledge was 14.5% and 78.7% respectively (Table 1).

Significant differences were found between levels of knowledge and levels of attitude towards COVID-19 (*P* < .001) and between the levels of knowledge and levels prevention practices (*P* < .001) among participants.

### 3.4. The attitude regarding COVID-19 among patients with type 2 diabetes

Most participants 232 (93.2%) strongly agreed that COVID-19 is a serious disease and 181 (72.7%) strongly agreed that COVID-19 is a curable disease. 230 (92.4%) and 232 (93.2%) of study participants reported that their chance -as patients with type 2 diabetes- of getting COVID-19 is high and they are more vulnerable to the unfavorable effects of COVID-19 than other people, respectively. 197 (79.1%) of respondents strongly agreed the concerned body should quarantine COVID-19 patients in a special quarantine center and 224 (90.0%) highly agreed that early detection of COVID-19 can enhance treatment outcome. COVID-19 disease concealing and COVID-19 home management were considered strongly disagreed by 220 (88.4%) and 93 (37.3%) respondents, respectively (Table 3).

**Table 3:** Attitude toward COVID-19 among patients with type 2 diabetes attending Abdullah-Khalil diabetes center, Sudan (n = 249).

Attitude-related questions	Responses				
	Strongly agree (N, %)	Agree (N, %)	Neutral (N, %)	Disagree (N, %)	Strongly disagree (N, %)
Did you believe that COVID-19 is a serious disease	232 (93.2)	12 (4.8)	4 (1.6)	0 (0)	1 (0.4)
Do you think that you will manage to carry out preventive measures recommended by the authority	155 (62.2)	48 (19.3)	17 (6.8)	15 (6.0)	14 (5.6)
Do you think that early detection of COVID-19 can enhance treatment outcome	224 (90.0)	14 (5.6)	10 (4.0)	0 (0)	1 (0.4)
Do you believe that COVID-19 can be managed at home	25 (10.0)	62 (24.9)	40 (16.1)	29 (11.6)	93 (37.3)
Do you agree that COVID-19 is a curable disease	181 (72.7)	46 (18.5)	15 (6.0)	3 (1.2)	4 (1.6)
Do you agree that COVID-19 results in death	230 (92.4)	12 (4.8)	6 (2.4)	0 (0)	1 (0.4)
The concerned body should quarantine COVID-19 patients in a special quarantine center	197 (79.1)	19 (7.6)	16 (6.4)	9 (3.6)	8 (3.2)
Do you think your chance-as patient with type 2 diabetes-of getting COVID-19 is high	230 (92.4)	10 (4.0)	9 (3.6)	0 (0)	0 (0)
Do you think patient with diabetes is more vulnerable to the unfavorable effects of COVID-19 than other people	232 (93.2)	9 (3.6)	8 (3.2)	0 (0)	0 (0)
Do you think COVID-19 patient should not talk about his case	7 (2.7)	2 (0.8)	6 (2.4)	14 (5.6)	220 (88.4)

Most of the participants 243 (97.6%) had positive attitude towards COVID-19 (Table 1).

The attitude levels had statistically significant association with levels of prevention practices towards COVID-19 (*P* = .005).

### 3.5. Preventive practice regarding COVID-19 among patients with type 2 diabetes

The mean ± SD score was 8 ± 1.7 for practice regarding COVID-19. 230 (92.4%) study participants reported that they will call the emergency hotlines or visit the health facility if they suspect infection with COVID-19. 161 (64.7%) participants had avoided large gathering and 201 (80.7%) had avoided going out of their homes to prevent spreading of COVID-19. Washing of hands with soap and water for at least 20 seconds, wearing of face mask when going out or in crowding areas and covering the mouth and nose during coughing and sneezing encountered by 230 (92.4%), 215 (86.3%), and 241 (96.8%) participants, respectively. Only 150 (60.2%) participants disinfect the frequently touched objects and surfaces (Table 4).

**Table 4:** Preventive practice measures regarding COVID-19 among patients with type 2 diabetes attending Abdullah-Khalil diabetes center, Sudan (n = 249).

Practice-related questions	Responses	
	Yes (N, %)	No (N, %)
If you suspecting infection with COVID-19, would you call the emergency hotlines or visit the health facility	230 (92.4)	19 (7.6)
Do you avoid going out of your home to prevent spreading of COVID-19	201 (80.7)	48 (19.3)
Do you wash your hands often using soap for at least 20 s	230 (92.4)	19 (7.6)
Do you wear face mask when going out or in crowding areas	215 (86.3)	34 (13.7)
Do you cover your mouth and nose during coughing and sneezing	241 (96.8)	8 (3.2)
Do you disinfect frequently touched objects and surfaces	150 (60.2)	99 (39.8)
Do you avoid large gathering	161 (64.7)	88 (35.3)
To prevent contracting COVID-19, do you take fruits and vegetables	222 (89.2)	27 (10.8)
To prevent contracting COVID-19, do you use herbal products and traditional medicine	183 (73.5)	66 (26.5)
Do you visit your doctor or health care facility frequently similar to before the report of COVID-19 in Sudan	171 (68.7)	78 (31.3)

COVID-19 affected the regular visits to the health care facility of 78 (31.3%) participants. The herbal products and traditional medicine used by 183 (73.5%) participants to prevent contracting COVID-19, while 222 (89.2%) participants took fruits and vegetables for prevention (Table 4).

### 3.6. The overall COVID-19 preventive practice

Data analysis showed that, the prevalence of poor COVID-19 preventive practice among the study participants was 10.4 % whereas the prevalence of moderate and good practice were 19.7% and 69.9% respectively (Table 1).

### 3.7. Association between the baseline characteristics and the level of knowledge, attitude, and preventive practices

Chi-square test and fisher exact test for association was used. The study participants were categorized into 3 categories for knowledge and practice: good, moderate and poor. And into 2 categories for attitude: positive and negative. Statistically significant association were found between the educational level and the level of knowledge (*P* < .001) showing high percentage of good and moderate knowledge among participants studied in secondary school or university. However, most participants with poor knowledge were illiterate (*P* < .001). Regarding attitude, high number of participants with of positive attitude were found in participants studied in secondary school or university, and negative attitude was found high in illiterate participants (*P* = .008) (Table 5).

**Table 5:** Association between the baseline characteristics and the level of knowledge, attitude and preventive practices among patients with type 2 diabetes attending Abdullah-Khalil diabetes center, Sudan (n = 249).

Characteristics	Level of knowledge			*P* value^†^	Attitude		*P* value^†^	Level of practice			*P* value^†^
	Poor, N = 17*	Moderate, N = 36*	Good, N = 196*		Negative attitude, N = 6*	Positive attitude, N = 243^*^		Poor, N = 26*	Moderate, N = 49*	Good, N = 174*	
Age group of the patient											
18–40 yr	0 (0.0%)	8 (22.2%)	44 (22.4%)	.094	0 (0.0%)	52 (21.4%)	.6	6 (23.1%)	8 (16.3%)	38 (21.8%)	.5
41–60 yr	9 (52.9%)	18 (50.0%)	105 (53.6%)		4 (66.7%)	128 (52.7%)		11 (42.3%)	25 (51.0%)	96 (55.2%)	
Above 60 yr	8 (47.1%)	10 (27.8%)	47 (24.0%)		2 (33.3%)	63 (25.9%)		9 (34.6%)	16 (32.7%)	40 (23.0%)	
Gender of the patient											
Female	12 (70.6%)	22 (61.1%)	98 (50.0%)	.2	3 (50.0%)	129 (53.1%)	>.9	13 (50.0%)	20 (40.8%)	99 (56.9%)	.13
Male	5 (29.4%)	14 (38.9%)	98 (50.0%)		3 (50.0%)	114 (46.9%)		13 (50.0%)	29 (59.2%)	75 (43.1%)	
Education level											
Illiterate	14 (82.4%)	14 (38.9%)	18 (9.2%)	**<.001**	4 (66.7%)	42 (17.3%)	**.008**	5 (19.2%)	9 (18.4%)	32 (18.4%)	.391
Khalwa (Holy Quran Institute)	1 (5.9%)	3 (8.3%)	8 (4.1%)		1 (16.7%)	11 (4.5%)		1 (3.8%)	0 (0.0%)	11 (6.3%)	
Primary school	2 (11.8%)	10 (27.8%)	58 (29.6%)		1 (16.7%)	69 (28.4%)		10 (38.5%)	14 (28.6%)	46 (26.4%)	
Secondary school	0 (0.0%)	5 (13.9%)	64 (32.7%)		0 (0.0%)	69 (28.4%)		4 (15.4%)	12 (24.5%)	53 (30.5%)	
University or above	0 (0.0%)	4 (11.1%)	48 (24.5%)		0 (0.0%)	52 (21.4%)		6 (23.1%)	14 (28.6%)	32 (18.4%)	
Marital status											
Divorced	0 (0.0%)	1 (2.8%)	10 (5.1%)	.3	0 (0.0%)	11 (4.5%)	.7	1 (3.8%)	3 (6.1%)	7 (4.0%)	.3
Married	14 (82.4%)	31 (86.1%)	163 (83.2%)		5 (83.3%)	203 (83.5%)		19 (73.1%)	40 (81.6%)	149 (85.6%)	
Single	1 (5.9%)	1 (2.8%)	17 (8.7%)		1 (16.7%)	18 (7.4%)		3 (11.5%)	5 (10.2%)	11 (6.3%)	
Widow	2 (11.8%)	3 (8.3%)	6 (3.1%)		0 (0.0%)	11 (4.5%)		3 (11.5%)	1 (2.0%)	7 (4.0%)	
Source of information (multiple responses question)											
Mass media	7 (41.2%)	28 (77.8%)	188 (95.9%)	**<.001**	2 (33.3%)	221 (90.9%)	**.001**	18 (69.2%)	45 (91.8%)	160 (92.0%)	**.006**
Social media	0 (0.0%)	4 (11.1%)	61 (31.1%)	**<.001**	0 (0.0%)	65 (26.7%)	.3	5 (19.2%)	15 (30.6%)	45 (25.9%)	.6
Family and friends	12 (70.6%)	27 (75.0%)	138 (70.4%)	.9	3 (50.0%)	174 (71.6%)	.4	17 (65.4%)	36 (73.5%)	124 (71.3%)	.8
Health care professionals	2 (11.8%)	3 (8.3%)	24 (12.2%)	.9	2 (33.3%)	27 (11.1%)	.15	2 (7.7%)	7 (14.3%)	20 (11.5%)	.7

Bold values means a statistically significant.

* Data were presented as Number and (percentage %).

Some source of information such as mass media had statistically significant association with the level of knowledge -which was found high among moderate and good knowledge groups-(*P* < .001), attitude-which was found high in positive attitude group-(*P* = .001), and preventive practices which was found high among moderate and good practice groups-(*P* = .006) among the study population. Other source of information such as social media was found only statistically significant with knowledge showing high percentage among good knowledge group (*P* < .001) (Table 5).

There was no statistically significant difference between male and female, different age groups or marital status in terms of knowledge, attitude, and preventive practices levels among the study participants (Table 5).

## 4. Discussion

Self and others protection according to the recommended protocols is an important thing for a highly contagious disease like COVID-19. Therefore, effective prevention through enhancing knowledge, attitude and preventive practices by high-risk persons in the community is an instant need.^[15]^

The characteristics of participants in this study were mainly female (53%), (28.1%) educated to primary school level, (83.5%) were married and (53.0%) within age group of (41–60) years. 25.7% of the participants have hypertension, 4.8% have cardiovascular diseases and 4.0% have pulmonary diseases as comorbidities.

The researcher found that, most of the participants (89.6%) reported the mass media (Television, Radio, Newspapers, Internet…etc) as the main source of information regarding COVID-19, while only (11.6%) of participants attained information via the health care professionals. This result was similar to Giao Huynh’ study in Vietnam and Rine’ study in Nigeria.^[15,16]^ Thus, it can be suggested that the patients are more interested in mass media than other forms, so the authorities in Sudan could use these findings to direct their efforts to inform the community regarding COVID-19 through this means.

The present study showed that 93.2% of participants had good and moderate level of knowledge towards COVID-19. This finding is relatively similar with that of Patrick Gad’(97%),^[17]^ Rine Christopher’ (99.5%),^[16]^ Rimesh’ (88%),^[14]^ and Zhi-Hao Li.^[18]^ This high proportion of knowledge among the study participants may be due to intensive awareness programs via mass media since the pandemic started till the study conducted, most of the participants (94.8%) aware of their high risk for COVID-19 as patients with type 2 diabetes and this may encourage them to know more, and educational level as more than 76.7% had got a formal education in different levels. However, the finding was higher than the study conducted in Sudan by Khawla Nasr Aldeen Altayb Mousa’^[19]^ May be due to that, it have been conducted in early pandemic era in Sudan and also in different study population.

The present study has shown indifference with the study done in Rwanda,^[17]^which has shown no statistically significant association between gender or age and knowledge score. Also shown indifference with the studies done in India, China, Hong Kong and Sudan,^[14,18–20]^ which have shown significant association between level of education and knowledge score. But the present study shown difference with the studies done in Sudan and India,^[14,19]^ which have shown association between marital status and knowledge score.

Concerning attitude, 93.2% of participants strongly believed that COVID-19 is serious disease. The majority of the respondents 92.4% reported that their chance -as patients with type 2 diabetes- of getting COVID-19 is high and they are more vulnerable to the unfavorable effects of COVID-19 than other people. 94% of the participants didn’t agree with concealing the COVID-19 cases in the society and dealing with it as stigma. The overall level of positive attitude towards COVID-19 among the participants was (97.6%) this finding may play an important role in control the COVID-19 pandemic. This finding is relatively similar to the studies conducted in Vietnam, Hong Kong, Rwanda and Sudan.^[15,17,19,20]^

This study showed that there is no association between age, gender or marital status and attitude of the participants towards COVID-19. But it explored significant association with the educational level and the source of information used by the participants.

Compared with the study conducted in India,^[14]^ the present study agrees with it that there is no association between the attitude and the marital status or gender, but it differs with it in the absence of the association with the educational level.

The prevalence of good prevention practices in this study was (69.9%). This result is relatively consistent with that of the cross-sectional study conducted among chronic illness patients in Vietnam (77.2%) and among the individuals with access to the internet during the national lockdown in north-central Nigeria (79.5%).^[15,16]^

This similarity may be due to high level of knowledge towards COVID-19 among the studies participants and high percent of them reported the mass media as main source of information regarding COVID-19, which contains the recommendations and updated news across the world regarding COVID-19 prevention.

This finding is better than that of the cross-sectional studies conducted in Ethiopia and Sudan,^[13,19]^ this could be due to the fact that this study was conducted at a later stage of the pandemic than them, when information about COVID-19 was found everywhere and accessible.

In the present study, there was no statistically significant association between the age, gender, education or marital status and the preventive practices score. This finding is not consistent with that of the studies conducted in (Vietnam, China, Hong Kong, Rwanda and Ethiopia).^[13,15,17,18,20]^

The possible reason for these differences might be due to a difference of study population and the concern related to the outbreak shared by this study participants (risk group).

This study found a significant association between the levels of attitude or prevention practices and knowledge score. Also, there was an association between the level of attitude and the prevention practices towards COVID-19. This findings are similar to the findings of the cross-sectional study conducted in Ethiopia.^[13]^

To the best of our knowledge, this is the first study assessing the knowledge, attitude and practices in selective disease populations (risk groups like patients with type 2 diabetes) towards COVID-19 amid the ongoing pandemic in Sudan. Also, we used an interview method for data collection rather than the online self-reported questionnaire. This may help in covering part of the population who has limited access to the internet, older adults or illiterates.

The study was conducted in 1 center of diabetes in Khartoum due to authorities’ restrictions during the pandemic. In addition, using of convenient sampling, these might not allow generalization of the results to other localities of Khartoum.

Khartoum state considered socioeconomically and educationally better than the other states, so the good level of knowledge, attitude and practice may not represent the underprivileged people outside it. Despite the assurance of confidentiality, the participants may give a socially acceptable answers in the interviews. Finally, no open-ended question was used, which may limit the information given.

## 5. Conclusion

Our study showed that most of patients with type 2 diabetes attending Abdullah-Khalil diabetes center had good knowledge, positive attitude and good prevention practices towards COVID-19. High educational level was found associated with good knowledge and positive attitude. However, participants with some source of information about COVID-19 had good knowledge and practice and positive attitude.

Despite good levels of knowledge, attitude and practices among the participant towards COVID-19 there’re still percent of poor knowledge and practices. Also, some misconceptions was noticed by the researcher among participants which need awareness raising programs directed to the patients with type 2 diabetes – a high-risk group – using mass media and in the centers via regular visits to improve their awareness.

## Acknowledgments

The assistance provided by the following doctors: Hayder Abu Ahmed, Ibtisam Shabbo, and Hani Sayed was greatly appreciated. Also, we would like to thank Abdullah-Khalil Center’ cadre and patients for their cooperation. The authors would like to thank the research agency: “Sudan Analytics for Research and Statistics” (https://www.facebook.com/Sudan.Analytics/, https://www.linkedin.com/company/76092484/, https://twitter.com/Sudananalytics, and https://telegram.me/Sudananalytics) for their help in our research.

## Author contributions

YSA: project administration, study and questionnaire design, data collection, data analysis and interpretation, writing manuscript and drafting the article. MIM: data collection. EAH: analysis plan, data analysis and interpretation, writing manuscript and drafting the article. ATO, IIA, SNB, and NEE: manuscript writing and editing. All authors revised the manuscript and approved it for publication.

**Conceptualization:** Yousra Ahmed, Alaa T. Omer.

**Data curation:** Yousra Ahmed, Meirfat I. Mohamed, Elfatih A. Hasabo.

**Formal analysis:** Yousra Ahmed, Elfatih A. Hasabo.

**Project administration:** Yousra Ahmed.

**Resources:** Yousra Ahmed, Meirfat I. Mohamed.

**Software:** Elfatih A. Hasabo.

**Supervision:** Elfatih A. Hasabo.

**Validation:** Yousra Ahmed.

**Visualization:** Yousra Ahmed.

**Writing – original draft:** Yousra Ahmed, Elfatih A. Hasabo.

**Writing – review & editing:** Yousra Ahmed, Elfatih A. Hasabo, Alaa T. Omer, Istabraq I. Abdelgadir, Sara N. Bashir, Noha E. EL Hag.
